# Cementitious Composites Reinforced with Waste Fibres from the Production of High-Quality Construction Textiles

**DOI:** 10.3390/ma15041611

**Published:** 2022-02-21

**Authors:** Ana Baričević, Katarina Didulica, Marina Frančić Smrkić, Marija Jelčić Rukavina

**Affiliations:** 1 Department of Materials, Faculty of Civil Engineering, University of Zagreb, 10000 Zagreb, Croatia; katarina.didulica@grad.unizg.hr (K.D.); marija.jelcic.rukavina@grad.unizg.hr (M.J.R.); 2 Department of Technical Mechanics, Faculty of Civil Engineering, University of Zagreb, 10000 Zagreb, Croatia; marina.francic.smrkic@grad.unizg.hr

**Keywords:** waste fibres, alkali resistant glass, basalt, carbon, mortar

## Abstract

In general, 20–25% of the original fibre weight is considered waste in the production of high-quality textiles for the construction sector. A market analysis has shown that in the Republic of Croatia alone, up to 327 tonnes of this waste is produced annually, which is enough to reinforce 50 to 150 thousand m^3^ of cementitious composites. This preliminary study aims to evaluate the contribution of glass, basalt and carbon fibres generated as waste in the local production of high-performance technical textiles, to the fresh and hardened properties of fibre reinforced mortars. In order to investigate the influence of fibres, three types of fibres in two different lengths (5 and 10 mm) were used, while the amount of fibres was constant. The obtained results show that due to the fibre presence, workability is reduced regardless of the type and length of the fibre. The tested fibres have a negligible effect on compressive strength, but the use of basalt and carbon fibres increases the tensile strength. Furthermore, all three types have positive influence on the toughness and volumetric deformations, although to a greater extent in the use of 10 mm long fibres and carbon fibres.

## 1. Introduction

Each structure, depending on its purpose, must be designed and constructed to meet the basic requirements for structures during their service life [1]. Unfortunately, experience has shown that a large number of concrete structures show signs of significant deterioration after only 20 to 30 years due to the interaction of mechanical and environmental factors [2]. The Organization for Economic Cooperation and Development (OECD) estimates that $7 trillion should be allocated annually to improve the world’s infrastructure [3]. Therefore, the funds invested in maintenance and repair today are higher than the cost of construction [4].

Nowadays, there are various techniques used for repairing and strengthening concrete structures. However, the most commonly used materials for the repair of concrete structures are fibre-reinforced cementitious composites. Depending on the intended use, these composites are used alone or reinforced with high quality textile fabrics. High quality textile fabrics are produced by various processing techniques such as spinning, weaving, knitting, lacing, loosening, needlework, and other modern techniques [5]. All techniques have in common that the starting material is a high-quality fibre, which may be glass, carbon, basalt or other types. Generally, 20–25% of the original fibre weight is considered to be production waste [6]. A small part of the waste is put into the furnace, melted and formed into, for example, glass beads, while in most cases this waste ends up in a landfill. Weaving of fabrics produces waste in two forms: (a) as direct roving ends and (b) as 40 to 60 mm wide net edges consisting of roving and thread.

UN Sustainable Development Goals call in their 9th goal to improve infrastructure and retrofit industry to make it sustainable by 2030, with increased resource efficiency and increased adoption of clean green technologies and industrial processes [7]. This is in line with the requirements of EU Regulation No. 305/2011 [8], which calls for the sustainable use of natural resources throughout the service life of a structure, with particular attention to the durability of structures and the use of environmentally friendly raw materials and secondary materials in structures. High value waste, such as this waste fibre, is a valuable resource and should be used for the same purpose for which it was originally produced, rather than ending up in a landfill. With the goal of using as little energy as possible in the recycling process, this waste can be turned into a valuable resource for the construction sector by shredding it. Especially since these same types of fibres are used every day to repair concrete structures. Typically, they are sold as dry mortar mixtures reinforced with short glass, basalt, carbon or other fibres.

The environmental benefits of using waste fibres are best illustrated by the amount of energy required to produce 1 kg of the fibres studied [9]: carbon fibres require 2.88 kg of water, 2.63 MJ of heat and 6.98 MJ of electricity; glass fibres 8.94 kg of water, 1.61 kWh of electricity and about 16 MJ of heat energy; basalt fibres the same amount of water 8.94 kg and about 18 MJ of heat and electricity. If the waste fibres are cut into short yarns before reuse and then interspersed in the mixtures, the estimated electricity consumption is about 0.00385 MJ, which is negligible. The use of waste fibres saves almost 100 per cent of the stated amounts of water and energy, and the impact on ozone layer depletion is minimal. The impact of waste fibres on global warming factor is also minimal as production of 1 kg of carbon fibres emits about 17 kg of CO_2_ and processing of waste fibres consumes about 0.00025 kg of CO_2_ per GJ of electricity (CO_2_ emissions from electricity—65.22 kgCO_2_/GJ) [10]. The use of waste fibres also contributes to the reduction of waste disposal as the resources are reused and/or recycled, ensuring efficient use of natural circular resources in line with the objectives of the economy.

The environmental and financial benefits should be further analysed, but preliminary analysis indicates an exceptional contribution especially since similar fibre types are used in cement composites for various reasons: to limit crack propagation, to increase ductility, to improve abrasion resistance, and for other reasons [11,12,13,14,15]. The choice of fibre type depends on the application of the final product. Various types of fibres are available in the market including synthetic and natural fibres. Nowadays, the specification, performance, production, and conformity of fibres for cementitious composites are strictly prescribed. Nevertheless, in recent years, new types of fibres have been studied and their application in concrete has been explored [16], as well as the use of waste/recycled fibres [17]. The list of potential waste fibres is constantly growing, but sufficient quantities must be available to ensure their future use in the construction industry. The market acceptance of high-performance textiles made from carbon, glass, or basalt fibres in industries such as construction, marine, automotive, wind energy, and others has contributed to a significant growth in their production. The global market for glass fibre reinforced concrete alone was estimated at $2.01 billion in 2018, while it is expected to reach $3.32 billion by 2023 [18]. At the same time, the carbon fibre market is expected to reach $8.9 billion by 2031 [19] and the basalt fibre market is expected to reach $397 million by 2024 [20]. A market analysis conducted as part of the ReWire project [21] showed that in the Republic of Croatia alone, up to 327 tonnes of this waste is generated annually, which is enough to reinforce 50 to 150 thousand m^3^ of cementitious composites. The waste is stored in containers and disposed of in a landfill. All this involves costs for the producer and at the same time is not in accordance with the Waste Directive [22]. This shows that there is a sufficient amount of locally available material that can be used for the production of construction materials. However, before their use in construction products, a detailed characterization of their properties is required to confirm a positive long-term behaviour, especially when exposed to an aggressive environment.

The research presented here aims to determine potential technological challenges that should be addressed in order to unlock the potential of waste fibres from the production of high-performance technical textiles and make them a valuable resource in the construction industry. So far, research has been mainly conducted on recycled composite products based on synthetic organic polymers reinforced with fibres [23,24,25,26,27], not on waste fibres from production. Therefore, this study presents a preliminary study on the potential use of alkali-resistant glass (GF), basalt (BF) and carbon (CF) waste fibres generated from the production of high-performance textiles for construction, as reinforcement in cementitious composites. In order to investigate the influence of fibres on the behaviour of cement composites, three types of fibres in two different lengths were used while the amount of fibres was constant. The effect was determined by examining the properties in the fresh (density, air content, consistency) and hardened state (compressive and flexural strength, total shrinkage, toughness).

## 2. Materials and Methods

### 2.1. Waste Fibres from the Production of High-Performance Textiles

The waste fibers used in the experimental study were produced by the Croatian company Kelteks Ltd., Karlovac, Croatia, which is one of the leading manufacturers of composite reinforcements in Europe. For more than 30 years Kelteks Ltd., Karlovac, Croatia, has been supplying many well-known distributors of structural and composite reinforcements in the world. In this research, alkali-resistant glass, basalt, and carbon fibers were used.

As explained earlier, textile production generates different types of fibre waste. This waste could represent a high value for the company because it can be transformed into high value products. The biggest challenge in using waste fibres in the construction industry is their inconsistent quality. Fortunately, waste fibres from the production of high-performance textiles are free of impurities, but to achieve the desired geometric properties and successful distribution in the cementitious composite, it is necessary to design the processing method of the fibres.

**Figure 1:**
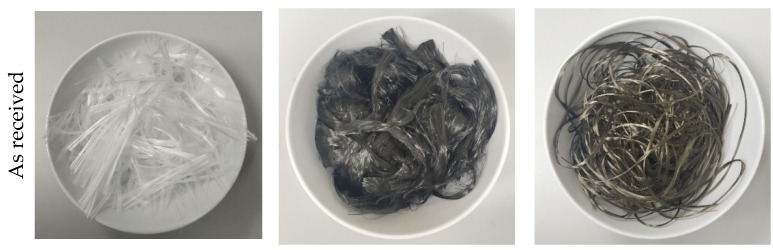

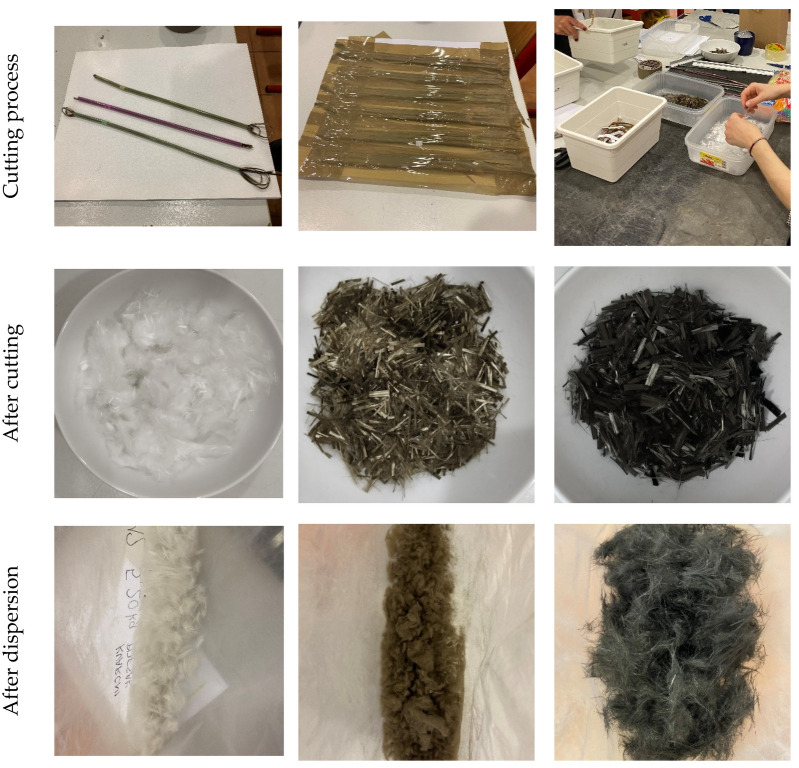
Visual appearance of the waste fibres used, from left to right: AR-glass, basalt and carbon fibres.

In this experimental study, the waste fibres came from the roving ends, which are a more elegant solution for processing compared to fibre edges with glass or viscous threads. In this study, each stage of processing was done manually, both cutting and dispersing the fibres. The fibres forming the end of the roving were first drawn into straws, which were then glued to technical paper and cut to the desired length with scissors (Figure 1). This is a time-consuming process as high-quality fibres are difficult to cut. In addition, the blade of the knife quickly becomes dull, making cutting even more difficult. Nevertheless, the fibres were cut in two lengths, namely 5 and 10 mm. The appearance of the fibres after cutting is shown in Figure 1.

The fibres used in this study are in yarns and therefore require additional dispersion prior to mixing. Dispersing ensures that the fibres are evenly mixed into the cementitious composite. To do this, the fibres are placed in a nylon bag and then subjected to 8 bar pressure for 3 min. The appearance of the fibres after dispersion is shown in Figure 1. Independently of this, a relatively high aspect ratio of the waste fibres can have a negative effect on their dispersion during mixing, especially if high fibre quantities are used.

Properties of fibres presented in Table 1 are received from the manufacturer. It can be assumed that the properties of the waste fibres may have changed slightly during the production process of fabrics and subsequent post-processing of the waste fibres compared to the original fibres.

The influence of fibre dimensions is evaluated using parameters such as fibre diameter, length, aspect ratio, reinforcement index, number of fibres per unit volume, and specific fibre surface area (Table 2). Aspect ratio is calculated as length over equivalent diameter and represents slenderness [15]. The reinforcement index is defined as the product of fibre volume fraction and aspect ratio [28], while the number of fibres describes the combined effect of fibre volume fraction, diameter, and length [15]. The fibre surface area represents a combined effect of fibre diameter and added volume fraction [15].

The ID of the particular mixture is as follows: GF_5 represents the mortar reinforced with 5 mm long alkali-resistant glass fibres, while GF_10 represents 10 mm long alkali-resistant glass fibres. The same was applied to basalt (BF) and carbon (CF) fibres, respectively.

### 2.2. Mix Design and Curing

The mortar was prepared using CEM I 42.5R, potable water and quartz sand with a maximum particle size below 1.3 mm. The mass ratio of cement: water: sand was 1:0.5:3. The chemical admixture, a polycarboxylate plasticizer, was added to each mixture to account for the effect of the fibres on workability. Additional amounts of superplasticizer were added to ensure uniform workability between mixtures (Table 3). However, all quantities used were below the maximum content prescribed by the manufactures, which is equivalent to 1% of the mass of the cement. Fibres of different types and lengths (5 and 10 mm) were added to a mixture in equal amounts, i.e., 5.5 kg/m^3^ of mortar.

In the mixing process, the dry components, i.e., cement, sand, and fibres, were mixed first. Water and a superplasticizer were then added. The fresh state properties were determined immediately after mixing. After casting, the specimens were covered under laboratory conditions for 24 h until demoulding to prevent water evaporation. After demoulding, the specimens were stored in the mist room at 20 ± 2 °C and RH ≥ 95% until they were tested at 28 days of age. Only the specimens for drying shrinkage measurements were stored under different conditions, namely at 20 ± 2 °C and RH ≥ 55% during the 90 days of ageing.

### 2.3. Methods

The properties of the mortar in fresh and hardened state were tested in accordance with the applicable standards, which are given in Table 4. Since the toughness test was not performed in accordance with a specific standard, the procedures described by other researchers were used [29,30,31], as detailed in the next paragraph. In the fresh state density, air content and consistency using the flow table were determined. At 28 days of age, compressive strength, flexural strength, and toughness were tested. Total shrinkage was measured from one up to 90 days of age. All tests were carried out on prismatic specimens with dimensions 40 mm × 40 mm × 160 mm.

#### Toughness

**Figure 2:**
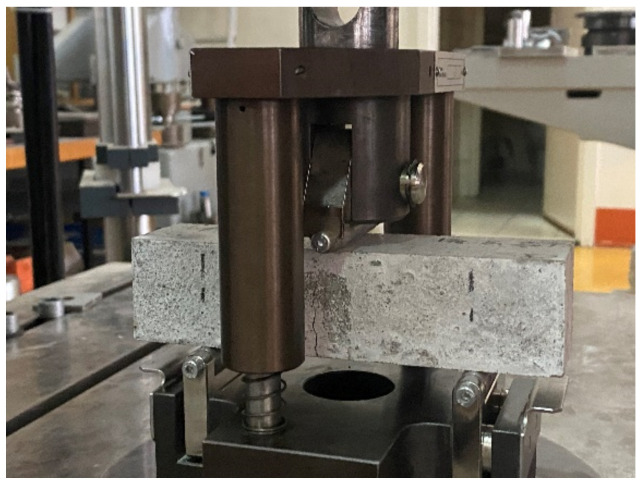
Toughness testing; positioning of the specimens and testing frame.

Three-point bending tests were performed on prism specimens with dimensions 40 mm × 40 mm × 160 mm (Figure 2). The specimens were simply supported with a span of 100 mm on steel rollers with a diameter of 10 mm. One of the support rollers and the loading roller can tilt slightly to allow uniform distribution of the load across the width of the prism without torsional stresses. The load was applied using a Zwick testing machine with a load capacity of 600 kN. The test was performed under displacement control at a rate of 0.05 mm/min up to a deflection of 0.2 mm and at a rate of 0.1 mm/min until the end of the test. This test rate was chosen to obtain a stable part of the curves after the peak and to obtain a localised crack in the middle of the span.

The peak value of the load-displacement curve represents the flexural strength of the specimens, and the effectiveness of the fibres is represented by the post-peak part of the curves. The energy absorption capacity, also called toughness, is the area under the load-displacement curve. The specific energy absorption capacity is determined by dividing the energy absorption capacity by the cross section of the specimen. Since the increase in energy absorption capacity after a 95% decrease in maximum load was negligible, the upper limit of integration was chosen at the deflection corresponding to this point.

## 3. Results and Discussions

### 3.1. Fresh State Properties of Mortar

The results of testing the properties of fibre-reinforced mortar in the fresh state are shown in Table 5.

In general, fibres affect the workability of the mixture; the greater the aspect ratio and the fibre amount, the more the consistency is reduced [32,33,34]. This behaviour is attributed to the larger surface area that needs to be wetted [34,35]. From Table 5 it can be seen that the results obtained in this work are in agreement with the literature, i.e., the consistency determined using the flow table decreased for mixtures with fibres compared to the reference mixture and the decrease was more pronounced for the fibres with larger aspect ratio and higher number of fibres per mm^2^.

The consistency of the mixtures with glass, basalt and carbon fibres was reduced by an average of 14, 26 and 26%, respectively, compared to the reference mixture. It should be emphasised that these results were obtained by increasing the superplasticizer content in the mixtures with basalt and carbon fibres by 13 and 65%, respectively, compared to the reference mixture (Table 3). The addition of superplasticizer brought all the considered mixtures to a similar consistency. The boundary between rigid and plastic consistency is at a flow table diameter of 140 mm [36].

The change in the properties of the fresh mortar due to the addition of fibres can be considered as a consequence of two basic parameters: (a) the aspect ratio, i.e., the ratio of fibre length to diameter, and (b) the proportion of fibres in the mixture [32,33,34,37,38]. These two parameters together are reflected in the fibre reinforcement index. It has already been shown that an increase in reinforcement index has a negative effect on workability [39]. The same trend was confirmed by the results presented here. The reinforcement index for glass and basalt fibres are 0.5 and 0.6, 1.0 and 1.2 for fibre lengths of 5 and 10 mm, respectively. The same index for carbon fibres is 2.1 and 4.3, respectively. The increase in the described index decreases the workability of the mixture.

The influence of fibres on properties in the fresh state is explained by the ability of fibres to absorb water and/or by the incorporation of needle-shaped constituents with large specific surface area into the composition [32,34,40]. Water absorption is mainly attributed to natural fibres, while synthetic fibres made of inorganic materials (carbon, glass, basalt, etc.) absorb small quantity or no water. Despite the changes in rheological behaviour, according to the work of Panzer et al. [32], no major changes are expected during the hydration process of cement composites with non-water absorbing fibres. When comparing glass and basalt fibres, the dominant influence is the higher absorption capacity of basalt fibres [41], which manifests itself in an average 14% lower consistency of the mixture with a similar reinforcement index. On the other hand, the question arises whether this absorbed water can be used later as an internal curing agent. However, the results presented here show that the type of inorganic fibres does not play a significant role in the deterioration of the properties in the fresh state, but rather the influence of the geometrical characteristics.

**Figure 3:**
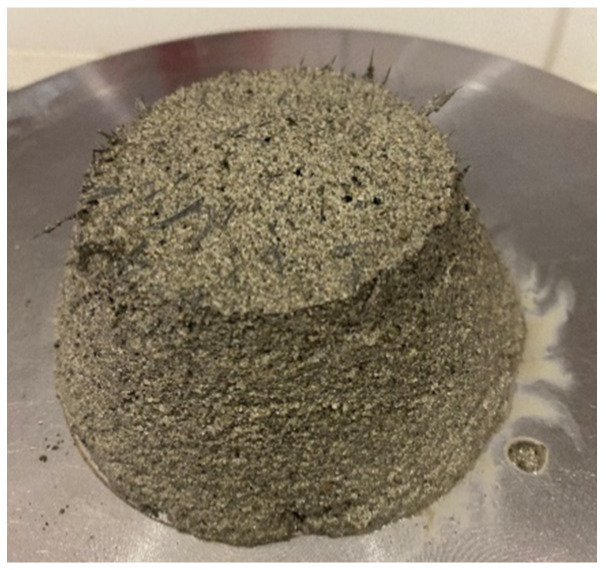
Improper dispersion of carbon fibres in fresh mortar.

It should be emphasised that the distribution of fibres in the cementitious composite depends precisely on the properties in the fresh state and is one of the most important influences on the properties in the hardened state. In particular, an improper dispersion of fibres in the fresh state was observed in a mixture with carbon fibres (Figure 3), which indicates the importance of developing appropriate technologies that lead to dispersion of the existing yarn into a single fibre. The dispersion of fibres in the mixtures is directly related to the number of individual fibres in the cementitious composite. As shown in Table 1, the number of filaments in yarn for carbon fibre is 47,000, while the same property is 1500 for glass fibre and 1000 for basalt fibre.

Despite this trend, it is interesting to note that the average reduction in air content in the fresh mortar for glass, basalt and carbon fibres is 16, 44 and 38%, respectively, compared to the reference mixture. This is reflected in the density of the fresh mortar, such that the density of glass fibre mortar is on average 4% higher, while the same property for basalt fibre and carbon fibre mortar is on average 9% higher than the reference mixture. The achieved decrease in density can be attributed to the higher amount of superplasticizer, which removes the surface tension of the water surrounding the molecules, increasing the flowability of the mortar and paving the way for the partial deagglomeration of the fibres in the fresh state [42]. Better dispersion, which could be further improved in the case of CF, results in fewer pores in the mixture and provides a better interface between the fibre and the matrix, which translates into a higher mixture density [43].

Here, the matrix had a strong effect on the porosity of the fibre reinforced composites, as the reference mixture already had significant porosity. Therefore, the presence of fibres had a positive effect, which is contrary to the previous studies [23,32,44]. Such high porosity is attributed to the used granulometry, and the rough surface of the quartz sand used. In further studies, the sand should be combined with an aggregate such as dolomite or similar, which has a higher content of fine particles to ensure adequate flowability and lower air content when fresh.

In addition, the fresh properties of fibre composites are also affected by the orientation and dispersion of the fibres. Insufficient dispersion is an obstacle to good compactness, porosity and density [35]. Consequently, it affects the mechanical properties in the hardened state [30]. In order to obtain high-strength fibre reinforced mortars with waste fibres, an integration technology that successfully curbs balling is required. Today, the dispersion of the fibres in the cementitious composites is ensured by various techniques. To improve the hydrophilicity of the fibres, various surface treatments of the fibres are studied before their incorporation into the cementitious mixture, such as ozone treatment, treatment with acidic and alkaline solutions, addition of silane or sizing with silane [30]. Fine particles, such as silica dust, are used to separate the fibres during mixing [45,46], just as in water-based polymer solutions. However, it is a technological challenge to develop advanced methods for processing and incorporating waste fibres into cementitious composites that are acceptable in daily practise. The waste fibres studied in this paper are ideal candidates for use in dry mix products. Therefore, dispersion techniques based on the combination of fine particle effect and air compression will be investigated in the future.

### 3.2. Hardened Properties of Mortar

#### 3.2.1. Compressive and Flexural Strength

**Figure 4:**
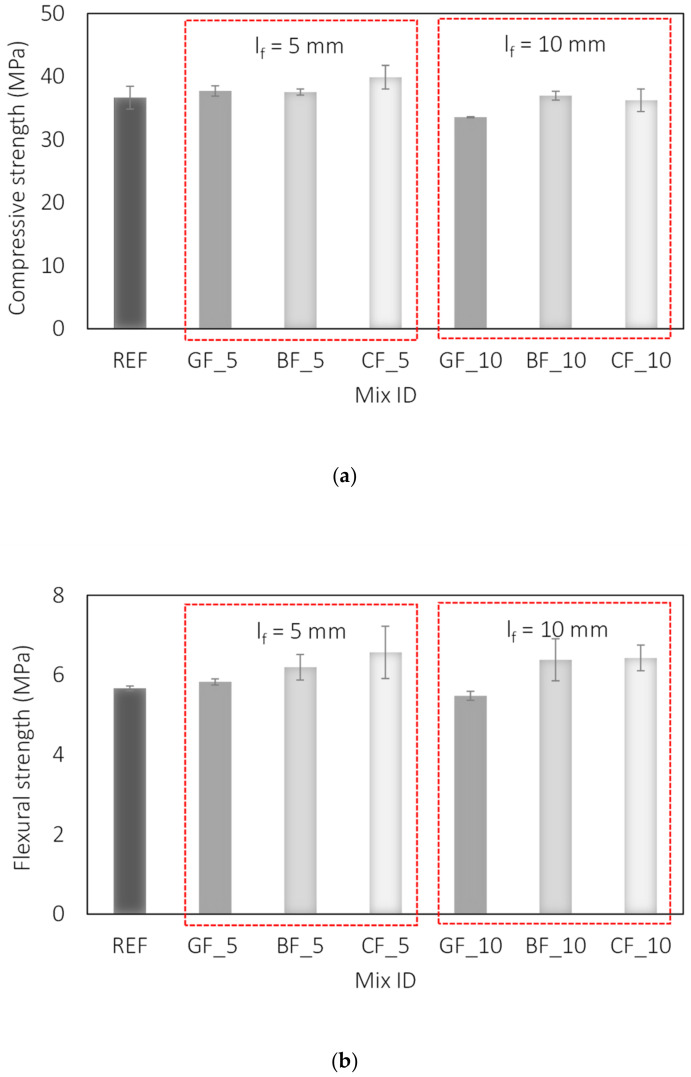
Influence of fibre type and length on: (**a**) compressive strength, (**b**) flexural strength of fibre reinforced mortar.

The results of the compressive strength of the tested mortars are shown in Figure 4a. It has been shown previously that the addition of microfibres helps to improve the fracture mechanism of the mortar, but has no significant effect on the value of compressive strength [38,47,48,49]. A similar trend was observed in the present study. Comparing the results of compressive strength (Figure 4a) and air content (Table 5), it could be concluded that the observed increase in compressive strength is due to the lower air content in the mixture and not to the presence of fibres. Therefore, it can be assumed that the types and lengths of fibres studied have a negligible effect on the compressive strength of fibre reinforced mortars.

These results are consistent with previous studies on glass and basalt fibre reinforced mortars [50,51,52], while for carbon fibre reinforced mortars they are consistent with [30] but not with [53,54]. The effect of the fibres on the compressive strength is explained by the greater air content and the balling effect of the fibres, which blocks the stress distribution. Moreover, due to the low content, the fibres cannot be effective as aggregate to resist the compression [50,52,55]. In this study, all types of fibres had a positive effect on the air content and compressive strength value, which can be attributed to the higher quantities of superplasticizer used and the associated lower air content compared to the reference mixture.

A greater influence of the fibres on the values of flexural strength of mortar [50,51,52,53,54] and also on the values of concrete [55,56] is expected. The influence of fibres on flexural strength lies in their high tensile strength values and their ability to resist tensile forces [53]. Furthermore, the contribution of fibres to the flexural strength of mortar is directly related to the properties of the fibres, such as length, diameter, tensile strength, and others [14,32]. Increasing the amount and length of fibres can improve the flexural strength up to a certain point. After that, no further improvement can be expected, or even a deterioration of the investigated properties occurs [12,49,57,58]. The results of flexural strength for tested mortars are given in Figure 4b showing that flexural strength increases with all three types of fibres. Here, an average increase in flexural strength of 11 and 15% was observed for mortar reinforced with basalt and carbon fibres of both lengths, respectively, compared to the reference mixture. Glass fibres did not make a significant contribution. These results are consistent with previous studies [50,55].

#### 3.2.2. Toughness

**Figure 5:**
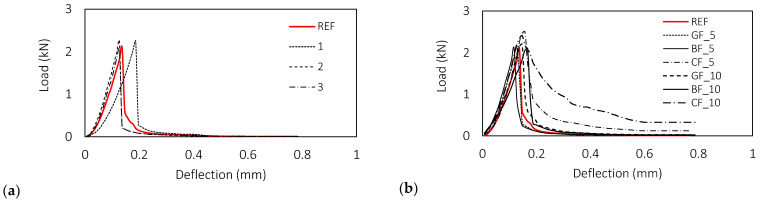

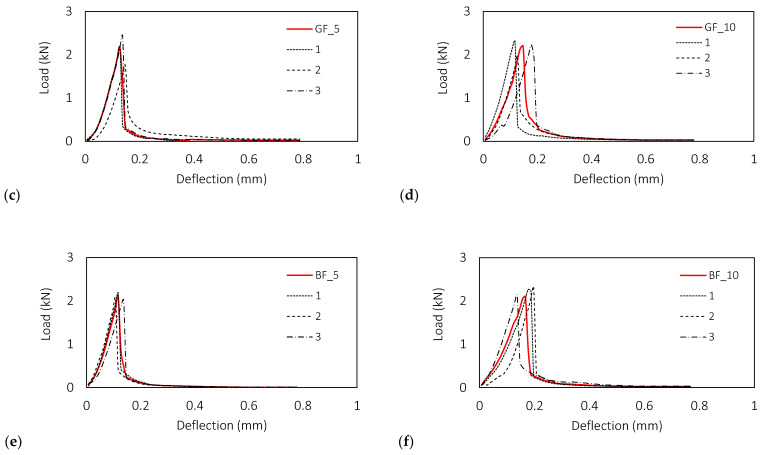

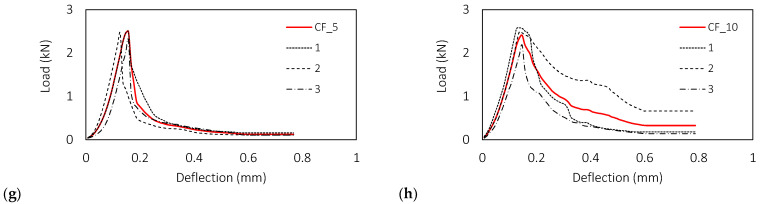
Influence of fibre type and length on toughness of fibre reinforced mortars: (**a**) referent mixture, (**b**) Average load—deflection curves, (**c**,**d**) glass fibres, (**e**,**f**) basalt fibres, (**g**,**h**) carbon fibres.

The obtained results of toughness testing of fibre reinforced mortars are presented in Figure 5 and show differences between types and fibre lengths. The load-displacement curves shown in Figure 5 confirm that only the carbon fibres contribute to the behaviour of the mortar in the post-cracking region. For a better understanding of the different contributions of the fibres, Table 6 contains the average values of maximum load and post-cracking load obtained at selected deflections i.e., 0.2, 0.3 and 0.4 mm.

In the analysis of maximum load, irrespective of their length, glass and basalt fibres do not contribute to the behaviour of the mortar before macrocracking. An increase of 7% in the maximum load was observed exclusively for carbon fibres of both lengths compared to the reference mixture (Table 6), which is in line with the flexural strength test results, Figure 4b.

**Figure 6:**
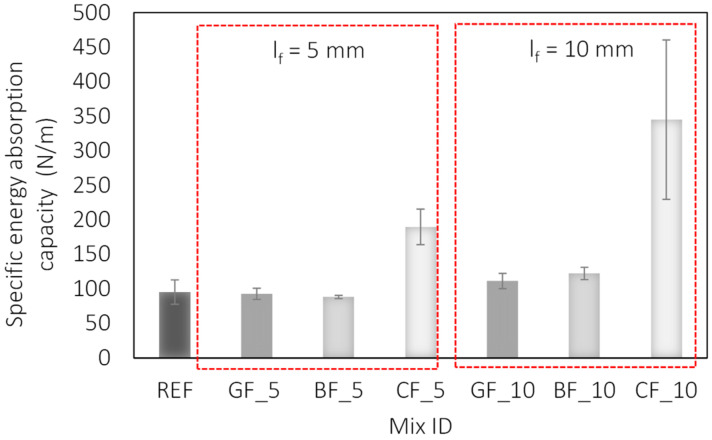
Influence of fibre type and length on specific energy absorption capacity of fibre-reinforced mortars.

The analysis of specific energy absorption capacities presented in Figure 6 confirms a significant contribution of carbon fibres for both tested lengths, registering an increase of 100 and 263% for fibres of length 5 and 10 mm with respect to the reference mixture. When analysing the different fibre lengths, an increase of 17, 28 and 263% in specific energy absorption capacity was observed for glass, basalt and carbon fibres with a longer length, i.e., 10 mm.

**Figure 7:**
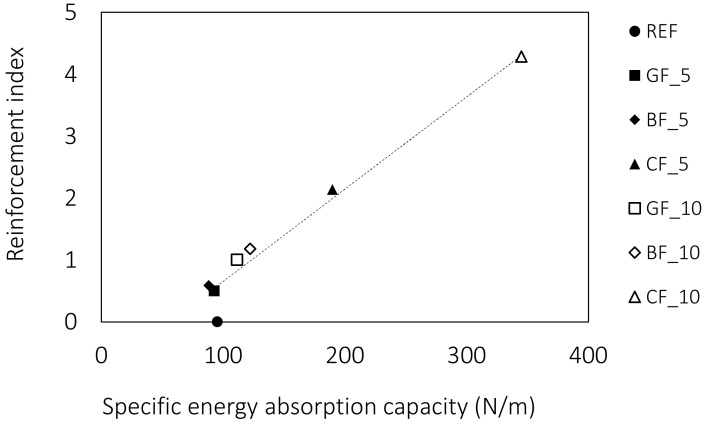
Correlation between the reinforcement index and specific energy absorption capacity.

The same trend is seen when the contribution of waste fibres to toughness is analysed. The greater influence of the carbon fibres can be attributed to the higher aspect ratio and thus the reinforcement index (Figure 7). From Table 1 and Table 2, which show the properties of the waste fibres, it can be seen that the reinforcement index and the number of fibres in the cross-section are significantly higher for carbon fibres. Due to the finer diameter, the number of individual carbon fibres is 6 and 16 times higher on average for selected fibre lengths. It is clear that the number of fibres bridging a potential crack theoretical increase when added in larger quantities, which translates into higher toughness.

**Figure 8:**
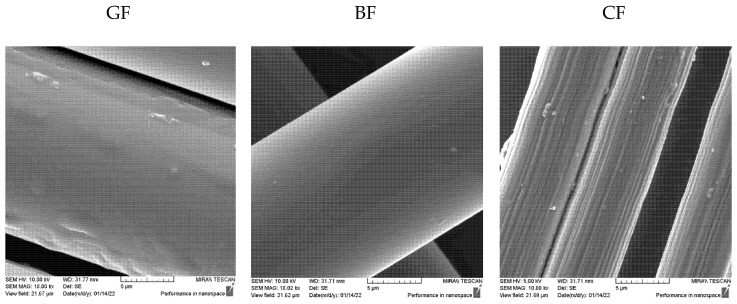
Surface properties of glass, basalt and carbon fibres photographed with a scanning electron microscope (10,000×).

The behaviour of cementitious composites in the post-cracking period is determined by the adhesion and friction at the interface between fibres and matrix, as well as by the resistance offered by the mechanical anchorage of the fibres in the composite [59]. Regarding adhesion, all the fibres studied are surface-sized, i.e., coated with a very thin polymer film to facilitate their handling and improve the mechanical performance of the composites. The sizing affects the hydrophilic behaviour of the fibres by increasing their surface energy and wettability [60,61]. The appearance of the surface of the waste fibres can be seen in Figure 8. The images taken with the scanning electron microscope show that the carbon fibres have the highest roughness, which could mean a better mechanism for transferring loads by friction. Although all the fibres studied exhibit sizing, only CF has wrinkles highlighted on the surface, while small “particles” on the surface, which could be caused by uneven coating of the sizing agent, are found on all surfaces. This is consistent with previous studies [62] showing that surface morphology is affected by the type of sizing agent. Here, the CF fibres have PU sizing agent, while both GF and BF contain silane sizing agents. The higher surface roughness of CF combined with the higher density and lower porosity of the mixture resulted in improved behaviour of the mortars reinforced with CF. According to [29], the higher compactness of the matrix and its lower porosity resulted in the fibre/matrix interfaces also having lower porosity, which in turn led to more fibre/matrix contact points and stronger interfacial bonding and stress transfer.

#### 3.2.3. Volumetric Deformations of Mortar

**Figure 9:**
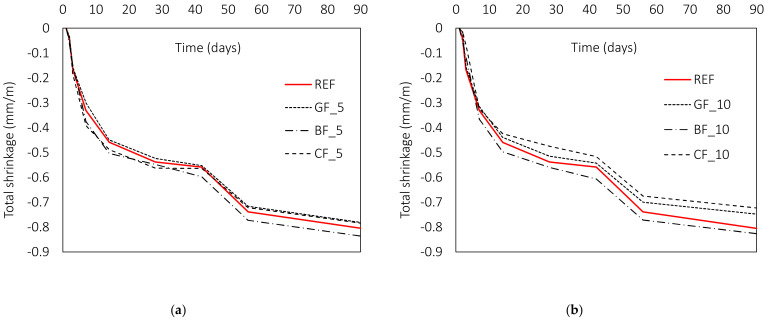
Influence of fibre type and length on total shrinkage of fibre-reinforced mortars: (**a**) l_f_ = 5 mm; (**b**) l_f_ = 10 mm.

The influence of fibres on volume deformations depends on both the geometrical and mechanical properties of the fibres. One of the most important contributions comes from the fibre modulus [15]. Indeed, the stiffness of the fibres should be higher than that of the matrix material to have a positive effect on cracking. This explains why low modulus fibres are often used to limit shrinkage cracking at an early age [15,39,63]. At this stage, the modulus of the fibres is still higher than the modulus of the matrix. In the present study, glass and basalt fibres have a lower modulus than carbon fibres, and all the fibres studied are microfibers (d ≤ 0.1 mm) [15]. Accordingly, the contribution of the studied fibres to the volumetric deformations is expected to be highest during first days after casting. During the hydration process, the volume of the composite decreases and microcracks form. This can be further controlled by microfibres with high tensile strength and high elastic modulus. According to the available literature, carbon [48], basalt [12,13,49,58] and glass fibres [13,49] show a reduction in shrinkage compared to a reference composite without fibres. However, the contribution of a particular type of fibre varies at different stages of its service life and depends not only on the type of fibre but also on its length. In the following, the results of the total shrinkage show minimal differences between the individual mixtures from the one to the 90-day period (Figure 9). However, analysis of the individual readings shows that the GF of both lengths and the CF with a length of 10 mm contribute to the values of total shrinkage during the first day. During this time, the shrinkage was reduced by an average of 21 (GF_5), 63 (GF_10) and 60% (CF_10) for the glass fibres and the carbon fibres, respectively. With time, the contribution of the fibres decreases with respect to the reference mixture, leading to an average reduction of 2, 5 and 9% for glass and carbon fibres. At the same time, the total shrinkage of mixtures with carbon fibres of 5 mm length and basalt fibres of both lengths is higher than the reference mixture by 7 (CF_5), 10 (BF_5) and 5% (BF_10), respectively.

**Figure 10:**
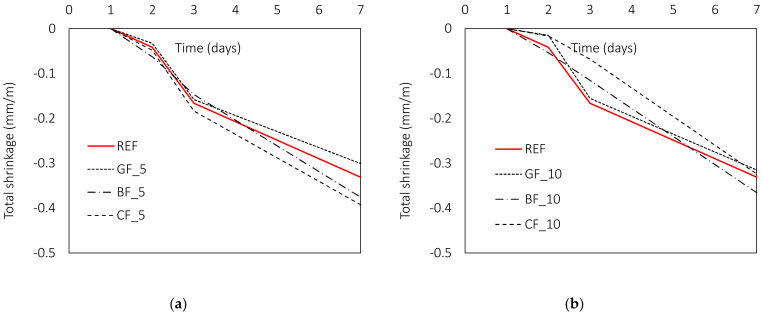
Influence of fibre type and length on early age total shrinkage: (**a**) l_f_ = 5 mm, (**b**) l_f_ = 10 mm.

**Figure 11:**
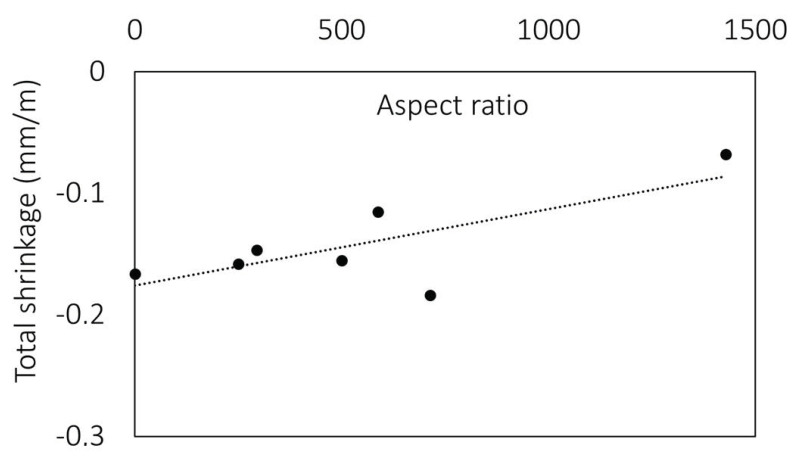
Influence of fibre aspect ratio on the total shrinkage values at the age of 3 days.

This trend can be seen in Figure 10, where the highest contribution during the first 3 days is made by the carbon fibres, followed by the basalt and glass fibres. The extent of their contribution to shrinkage reduction is directly related to the values of their diameter and elastic modulus (Figure 11), where carbon has 3.4 and 2.9 times higher modulus compared to basalt and glass fibres, respectively, while basalt has 1.9 times higher modulus than glass fibres. At the same time, the diameter of carbon is 2.5–3 times smaller than diameter of glass and basalt fibre. The described positive effect of fibres is observed only for mixtures with fibres of 10 mm length. For shorter fibres, this contribution is not observed in the total shrinkage values. This is in agreement with previous studies by [64].

As the aspect ratio of fibres increases, the shrinkage of composites with the same fibre content decreases (Figure 11). Due to the lower elastic modulus of the matrix, the effect of the fibres on the shrinkage of the cement matrix was more significant at an earlier age and therefore should be further investigated for the waste fibres used in the study. This is consistent with previous research by the authors [65], where it was shown that the use of waste microfibres improved the properties of cement composites at an early age.

## 4. Conclusions

Currently, waste fibres generated in the production of high-performance technical textiles are mostly discarded or used for energy purposes, although they have same or similar properties as those whose use in cement composites is common but often limited due to high production costs. From the study presented in this paper and carried out on the mortars made with glass, basalt and carbon fibres with different lengths, the following conclusions can be drawn:

– The presence of fibres has a negative influence on the workability of the mortar irrespective of the type and length of fibres.
– The type and length of fibres studied have a negligible effect on the compressive strength but lead to an average increase in flexural strength of 11 and 15% for basalt and carbon fibre reinforced mortars of both lengths, respectively, compared to the reference mixture. Glass fibres did not make any significant contribution.
– The analysis of specific energy absorption capacity confirms a significant contribution of carbon fibres, showing an increase of 100 and 263 for fibres with length of 5 and 10 mm, respectively, compared to the reference mixture. Higher reinforcement index provides further increase in specific energy absorption capacity by 17, 28 and 263% for glass, basalt, and carbon fibres with length of 10 mm compared to shorter fibres.
– The total shrinkage values are reduced for longer fibres (10 mm), more significantly at early ages, i.e., up to 3 days. The contribution of fibres correlates with their elastic modulus and aspect ratio; an increase in both properties leads to lower volumetric deformations. For shorter fibres (5 mm), this contribution is not observed in the total shrinkage values.

Although the results indicate beneficial behaviour of carbon fibres, the geometrical and mechanical parameters of each fibre type should be considered before selecting the most effective fibre type. The significantly lower linear density of the glass and basalt waste yarns used compared to carbon yarn may have contributed to their modest contribution, especially in toughness, and may be related to insufficient fibre dosage in the mixture. Further research is needed to characterize the waste fibres produced in the manufacture of high-quality textiles, but the initial results are promising. In order to fully exploit the potential of the waste fibres investigated, a high-performance matrix needs to be developed that both justifies the use of such high-quality fibres and meets the requirements of the relevant standards for repair materials. In addition, the resistance of these fibres in alkaline environments should also be the subject of future investigations.

## Figures and Tables

**Table 1:** Characteristics of waste fibres as obtained by the manufacturer.

Fibre ID	Filament Diameter [µm]	No. of Filaments in Yarn	Linear Density [tex]	Tensile Strength of Yarn [MPa]	Modulus of Elasticity of Yarn [GPa]	Sizing
Alkali-resistant glass (GF)	20	1500	1200	1000–1700	72	silane
Basalt (BF)	17	1000	600	2900–3100	86	silane
Carbon (CF)	7	47,000	3200	4300	250	PU

**Table 2:** Specific fibre characteristics calculated based on fibre properties.

Fibre Property	Fibre Type					
	* l_f_ = 5 mm			l_f_ = 10 mm		
	GF_5	BF_5	CF_5	GF_10	BF_10	CF_10
Aspect ratio	250	294	714	500	588	1429
Reinforcement index	0.5	0.6	2.1	1.0	1.2	4.3
Number of fibres per mm^2^	1.27	1.76	15.60	0.64	0.88	7.80
Specific fibre surface	0.40	0.47	1.71	0.40	0.47	1.71

* fibre length.

**Table 3:** Superplasticizer quantities depending on the type and quantity of fibres.

Property	REF	l_f_ = 5 mm			l_f_ = 10 mm		
		GF_5	BF_5	CF_5	GF_10	BF_10	CF_10
Superplasticizer [% per m_c_]	0.40	0.40	0.45	0.65	0.45	0.45	0.65

**Table 4:** Test methods for characterization of mortar properties.

Property	Standard	Number of Specimens per Mixture
Density	EN 1015-6:2000/A1:2008	-
Air content	EN 1015-7:2000	
Consistency by flow table	EN 1015-3:2000/A1:2005/A2:2008	
Compressive strength	EN 196-1:2016	6
Flexural strength	EN 196-1:2016	3
Total shrinkage	EN 12390-16	3
Toughness	-	6

**Table 5:** Fresh state properties of fibre reinforced mortars.

Property	REF	l_f_ = 5 mm			l_f_ = 10 mm		
		GF_5	BF_5	CF_5	GF_10	BF_10	CF_10
Density [g/cm^3^]	1833	1910	1991	2009	1909	1989	1971
Flow table r_average_ [mm]	156	135	116	119	133	115	111
Air content [%]	16.0	13.0	9.5	10.0	14.0	8.5	9.7

**Table 6:** The average of maximum loads and post-cracking loads at selected deflections.

Load Level	REF	l_f_ = 5 mm			l_f_ = 10 mm		
		GF_5	BF_5	CF_5	GF_10	BF_10	CF_10
F_max_ (kN)	2.28 ± 0.19	2.15 ± 0.3	2.15 ± 0.11	2.44 ± 0.07	2.20 ± 0.19	2.31 ± 0.11	2.44 ± 0.19
F_0,2_ (kN)	0.14 ± 0.11	0.17 ± 0.11	0.11 ± 0.02	0.76 ± 0.38	0.26 ± 0.12	0.30 ± 0.07	1.61 ± 0.52
F_0,3_ (kN)	0.06 ± 0.03	0.08 ± 0.07	0.04 ± 0.0	0.35 ± 0.08	0.10 ± 0.03	0.10 ± 0.03	0.95 ± 0.50
F_0,4_ (kN)	0.04 ± 0.02	0.05 ± 0.06	0.03 ± 0.01	0.24 ± 0.06	0.06 ± 0.01	0.06 ± 0.03	0.65 ± 0.57

## Data Availability

Not applicable.
