# Correction: Baričević et al. Cementitious Composites Reinforced with Waste Fibres from the Production of High-Quality Construction Textiles. *Materials* 2022, *15*, 1611

**DOI:** 10.3390/ma15113821

**Published:** 2022-05-27

**Authors:** Ana Baričević, Katarina Didulica, Marina Frančić Smrkić, Marija Jelčić Rukavina

**Affiliations:** 1 Department of Materials, Faculty of Civil Engineering, University of Zagreb, 10000 Zagreb, Croatia; katarina.didulica@grad.unizg.hr (K.D.); marija.jelcic.rukavina@grad.unizg.hr (M.J.R.); 2 Department of Technical Mechanics, Faculty of Civil Engineering, University of Zagreb, 10000 Zagreb, Croatia; marina.francic.smrkic@grad.unizg.hr

## Error in Figure

In the original publication [1], there was a mistake in ***Figure 11. Influence of fibre aspect ratio on the total shrinkage values at the age of 3 days*** as published. Data are missing on the figure and the equation should be omitted. The corrected ***Figure 11.*** appears below.

Published: 

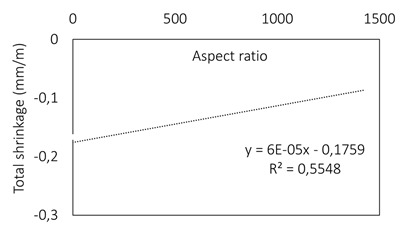



Corrected: 

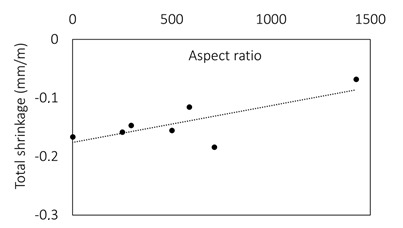



**Text Correction**

In the original publication, a correction has been made to ***2. Materials and Methods*, *2.1. Waste Fibres from the Production of High-Performance Textiles, First Paragraph*** as follows:

Published:

**For more than 30 years Kelteks Ltd., Karlovac, Croatia. Has been supplying many well-known distributors of structural and composite reinforcements in the world.**

Corrected:

**For more than 30 years Kelteks Ltd., Karlovac, Croatia, has been supplying many well-known distributors of structural and composite reinforcements in the world.**

In the original publication, a correction has been made to ***2. Materials and Methods*, *2.3. Methods, Toughness*** *as follows:*

Published: 

**The specimens were simply supported with a span of 100 mm on steel rollers with a radius of 10 mm.**

Corrected: 

**The specimens were simply supported with a span of 100 mm on steel rollers with a diameter of 10 mm.**

In the original publication, a correction has been made to ***3. Results and Discussions*, *3.2. Hardened Properties of Mortar, 3.2.2. Toughness, Second Paragraph*** as follows:

Published:

**An increase of 7% in the maximum load was observed exclusively for carbon fibres of both lengths compared to the reference mixture (Table 6), what is in line with the flexural strength test results, Figure 2.**

Corrected:

**An increase of 7% in the maximum load was observed exclusively for carbon fibres of both lengths compared to the reference mixture (Table 6), which is in line with the flexural strength test results, Figure 4b.**

In the original publication, a correction has been made to ***3. Results and Discussions*, *3.2. Hardened Properties of Mortar, 3.2.2. Toughness, Third Paragraph*** as follows:

Published:

**When analysing the different fibre lengths, an increase of 17, 28 and 263% in specific energy absorption capacity was observed for glass, basalt and carbon fibres with a longer length, i.e., 10 mm (Figure 5).**

Corrected:

**When analysing the different fibre lengths, an increase of 17, 28 and 263% in specific energy absorption capacity was observed for glass, basalt and carbon fibres with a longer length, i.e., 10 mm.**

The authors apologize for any inconvenience caused and state that the scientific conclusions are unaffected. This correction was approved by the Academic Editor. The original publication has also been updated.
